# Supplementary material for: Structural evolution of a DNA repair self-resistance mechanism targeting genotoxic secondary metabolites
Source: Nat Commun. 2021 Nov 26;12:6942. doi: 10.1038/s41467-021-27284-7 (PMC8626424; doi:10.1038/s41467-021-27284-7)
Supplement: Supplementary file 1 — Supplementary Information [file 41467_2021_27284_MOESM1_ESM.pdf]

SUPPLEMENTARY INFORMATION

Structural evolution of a DNA repair self-resistance mechanism targeting genotoxic secondary metabolites

Elwood A. Mullins[1,‡], Jonathan Dorival[1,‡], Gong-Li Tang[2], Dale L. Boger[3], and Brandt F. Eichman[1,4,*]

[1]Department of Biological Sciences, Vanderbilt University, Nashville, Tennessee, USA

[2]State Key Laboratory of Bio-organic and Natural Products Chemistry, Shanghai Institute of Organic Chemistry, Chinese Academy of Sciences, Shanghai, China

[3]Department of Chemistry, The Scripps Research Institute, La Jolla, California, USA

[4]Department of Biochemistry, Vanderbilt University School of Medicine, Nashville, Tennessee, USA

[‡]Contributed equally to this work

*Corresponding author: brandt.eichman@vanderbilt.edu

Supplementary Results

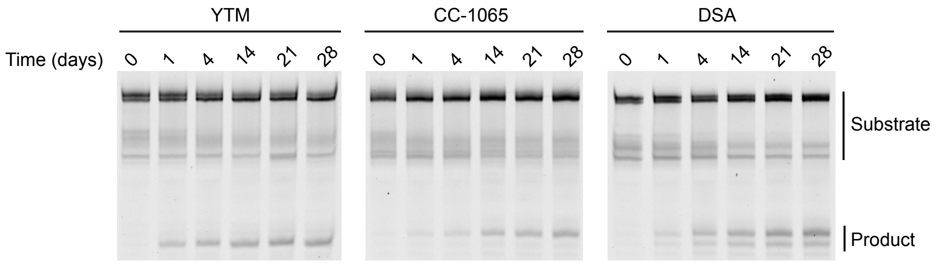

Supplementary Figure 1. Spontaneous depurination of SCPCHD adducts. Full-length DNA substrate (25-mer) and cleaved product (12- or 13-mer), generated by alkaline cleavage of the AP site, were separated by denaturing gel electrophoresis. The high melting temperature of the GC-rich substrate prevented complete denaturation of the DNA duplex, producing a smeared substrate band. Experiments were performed in triplicate.

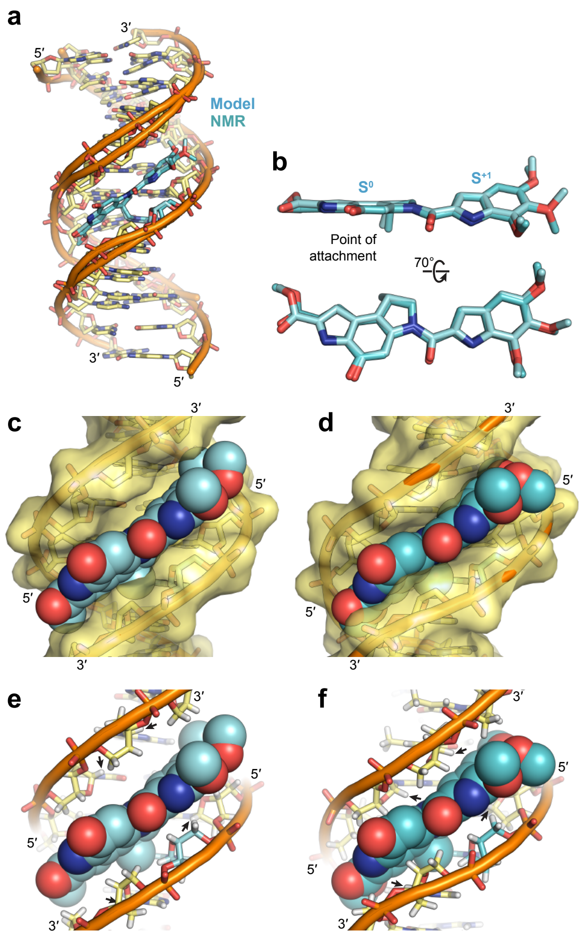

Supplementary Figure 2. Comparison of hypothetical and experimental structures of DSA-DNA. (**a**) Superposition of the hypothetical DSA-DNA model from this study (light cyan) and a previously published NMR structure (dark cyan) (PDB accession 1DSA). (**b**) DSA extracted from the model and the NMR structure. The point of attachment to the DNA is indicated. (**c**,**d**) Close-up views of the hypothetical DSA-DNA model (**c**) and the NMR structure (**d**). Relative to ideal B-form DNA, the minor groove around the adduct in the NMR structure is narrowed by ~1 Å. (**e**,**f**) Close-up views of the hypothetical DSA-DNA model (**e**) and the NMR structure (**f**) showing CH-π interactions between the DNA backbone and the DSA adduct. Narrowing of the minor groove creates stronger CH-π interactions, while rotation of the backbone directs the C-H bond at C1′ toward the adduct, increasing the number of CH-π interactions. In ideal B-form DNA, only the C-H bonds at C4′ and C5′ are appropriately oriented to form CH-π interactions with the DSA adduct. Re-oriented C-H bonds are indicated with arrows.

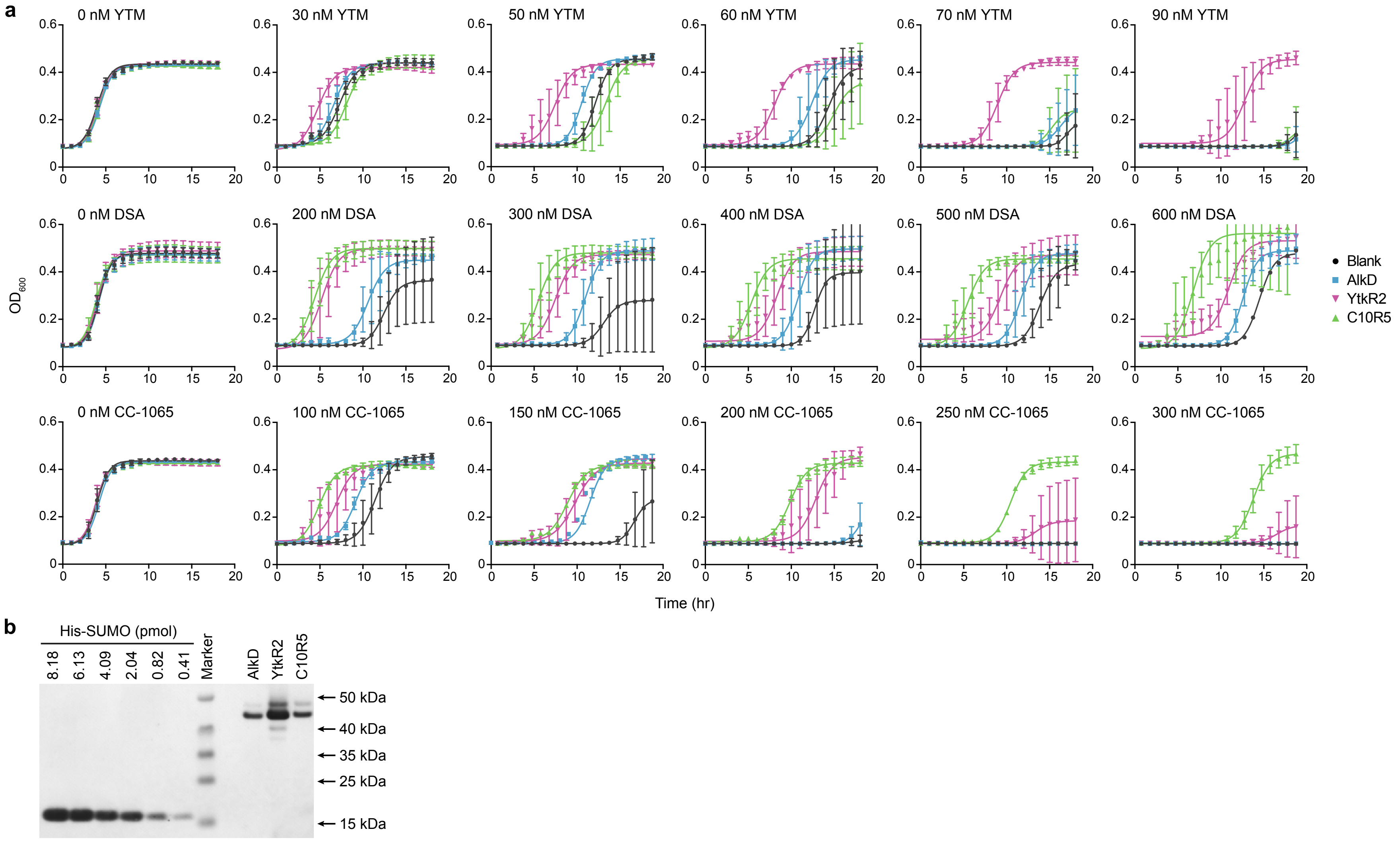

Supplementary Figure 3. SCPCHD resistance. (**a**) Growth curves. *E. coli* BL21(DE3) cells were transformed with either a plasmid encoding a hexahistidine-SUMO fusion construct of AlkD, YtkR2, or C10R5 or encoding only the hexahistidine-SUMO tag alone (blank). Cultures were grown in the presence of various concentrations of YTM, DSA, or CC-1065. Data are presented as the mean ± SD from four replicate experiments. Source data are provided as a Source Data file. (**b**) Western blot. Protein expression in *E. coli* BL21(DE3) precultures was detected using an anti-hexahistidine antibody. Band intensity was plotted against known amounts of pure hexahistidine-SUMO tag to determine absolute quantities of AlkD [2.1 ± 0.7 pmol; (9.8 ± 3.3) × 10^4 molecules/CFU], YtkR2 [5.1 ± 0.2 pmol; (2.4 ± 0.1) × 10^5 molecules/CFU], and C10R5 [2.4 ± 0.3 pmol; (1.1 ± 0.1) × 10^5 molecules/CFU]. Experiments were performed in triplicate.

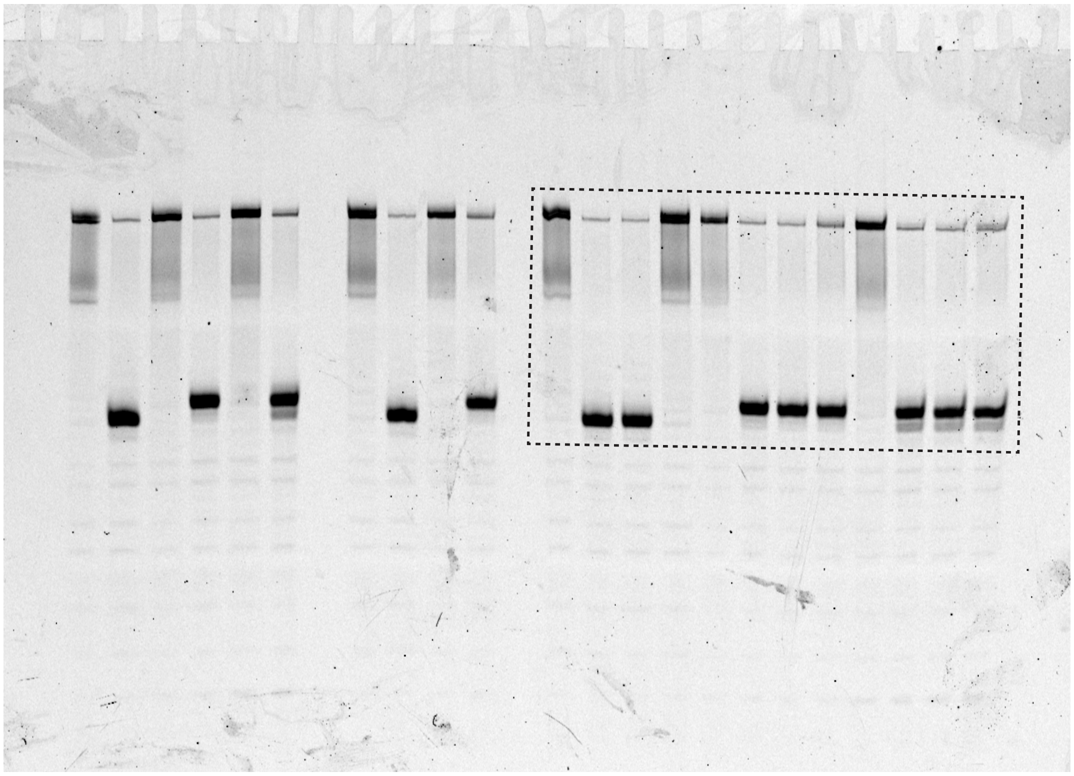

Supplementary Figure 4. Full gel image. The gel image in **Fig. 4b** was cropped from a larger image. The cropped area is indicated with a dashed box.

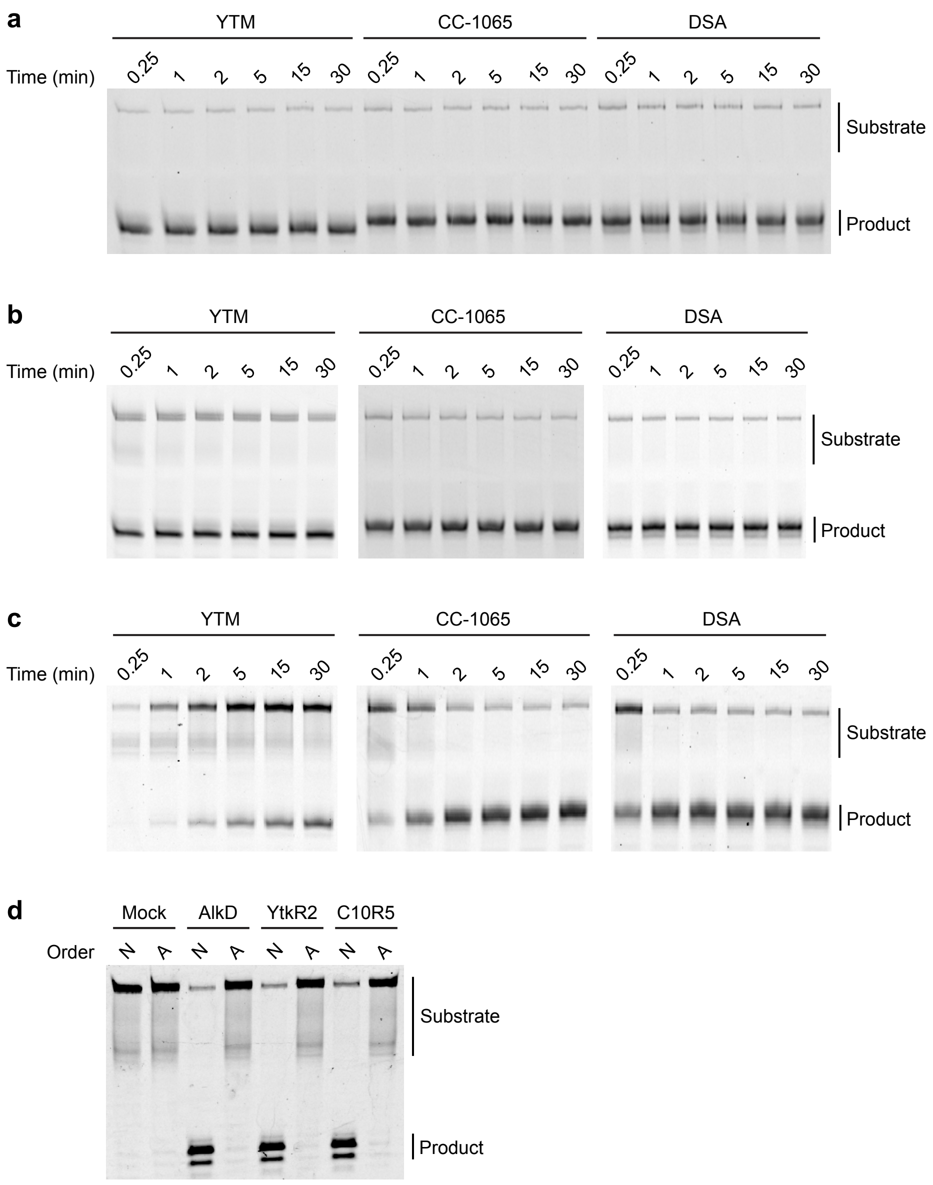

Supplementary Figure 5. Single-turnover excision of SCPCHD adducts. (**a**–**c**) Modified nucleobases in a defined oligonucleotide substrate were excised by AlkD (**a**), YtkR2 (**b**), and C10R5 (**c**). Reactions contained 1 μM enzyme and 100 nM DNA. Full-length DNA substrate (25-mer) and cleaved product (12- or 13-mer), generated by alkaline cleavage of the AP site, were separated by denaturing gel electrophoresis. The high melting temperature of the GC-rich substrate prevented complete denaturation of the DNA duplex, producing a smeared substrate band. (**d**) Confirmation of reaction quenching conditions. Normally (N), reactions are initiated by adding enzyme to substrate and then quenched by adding NaOH. Alternatively (A), to determine if product is generated after the alkaline quench, NaOH was added to substrate before enzyme was added. Both reactions proceeded for 0.25 min prior to addition of the final component. Reactions contained 100 nM DSA-DNA and either 1 μM enzyme or 0.25 M NaOH. Using the alternative order of addition, no product was observed with any of the homologs, beyond that seen in a no-enzyme control (mock), indicating the alkaline quench is both rapid and complete. All experiments were performed in triplicate.

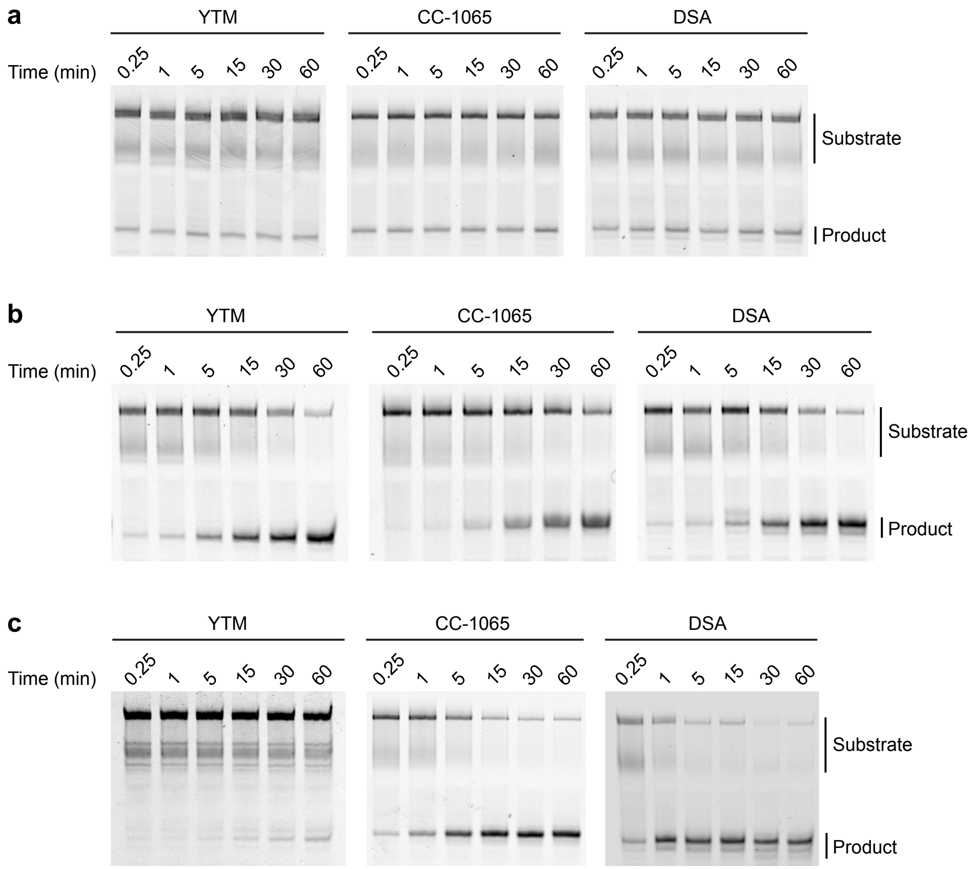

Supplementary Figure 6. Multiple-turnover excision of SCPCHD adducts. (**a**–**c**) Modified nucleobases in a defined oligonucleotide substrate were excised by AlkD (**a**), YtkR2 (**b**), and C10R5 (**c**). Reactions contained 10 nM enzyme and 100 nM DNA. Full-length DNA substrate (25-mer) and cleaved product (12- or 13-mer), generated by alkaline cleavage of the AP site, were separated by denaturing gel electrophoresis. The high melting temperature of the GC-rich substrate prevented complete denaturation of the DNA duplex, producing a smeared substrate band. All experiments were performed in triplicate.

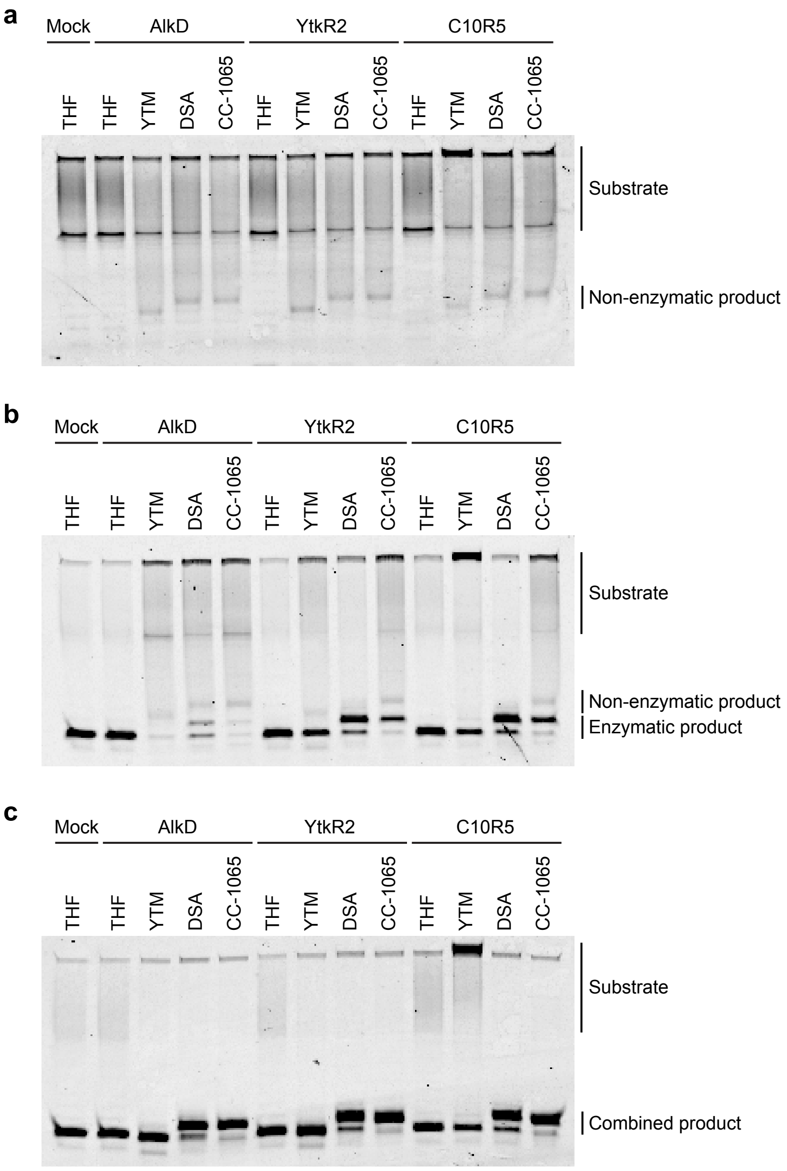

Supplementary Figure 7. Incision of AP-DNA. (**a**) Non-enzymatic cleavage of AP-DNA generated from each of the SCPCHD adducts. (**b**) Enzymatic incision of AP-DNA by EndoIV. (**c**) Alkaline cleavage of AP-DNA by NaOH. AP-DNA was generated from each of the SCPCHD adducts by pre-incubation with AlkD, YtkR2, or C10R5. Each of the homologs was also separately pre-incubated with THF-DNA and 3mAde nucleobase. Mock reactions contained THF-DNA and 3mAde but lacked enzyme. Aliquots from each of the primary reaction mixtures were added to secondary reaction mixtures with or without EndoIV. Quenching of the secondary reactions with EDTA (**a** and **b**) allowed for quantitation of both non-enzymatic and enzymatic AP-DNA cleavage, while quenching with NaOH (**c**) enabled quantitation of all AP-DNA present in the reaction mixtures, defining the upper limits for the AP-DNA incision reactions. THF-DNA was unaffected by NaOH. Full-length DNA substrate (25-mer) and cleaved product (12- or 13-mer) were separated by denaturing gel electrophoresis. The high melting temperature of the GC-rich substrate prevented complete denaturation of the DNA duplex, producing a smeared substrate band. All experiments were performed in triplicate.

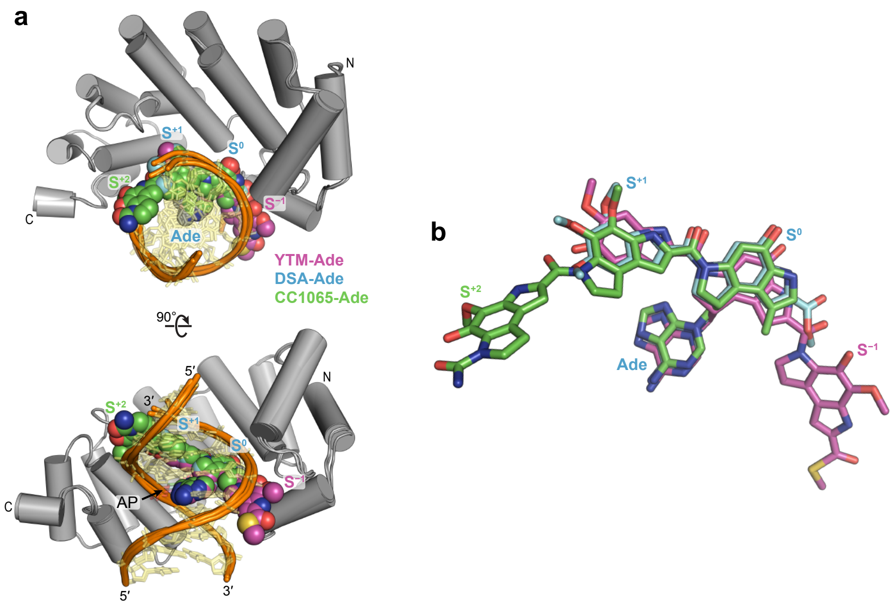

Supplementary Figure 8. Superposition of AlkD product complexes. (**a**) AlkD complexes containing AP-DNA and either YTM-Ade, DSA-Ade, or CC1065-Ade. Subunits S^{-1} of YTM and S^{+2} of CC-1065 partly extend beyond the substrate binding surface of AlkD. (**b**) SCPCHD adducts extracted from the AlkD product complexes. Relative to DSA-Ade and CC1065-Ade, contacts between AlkD and subunit S^{-1} of YTM-Ade rotate the adduct within the minor groove, pushing the S^{-1} and S^{0} subunits toward the DNA and the S^{+1} subunit toward the protein. This rotation creates a hydrophobic contact between the methoxy group on subunit S^{+1} and Pro117 that is absent in the other AlkD product complexes. In YtkR2 and C10R5, this position is occupied by glycine and leucine residues, respectively. All attempts to determine the effect of a possible steric clash involving this position (Leu122) in C10R5 through mutational analysis produced insoluble protein.

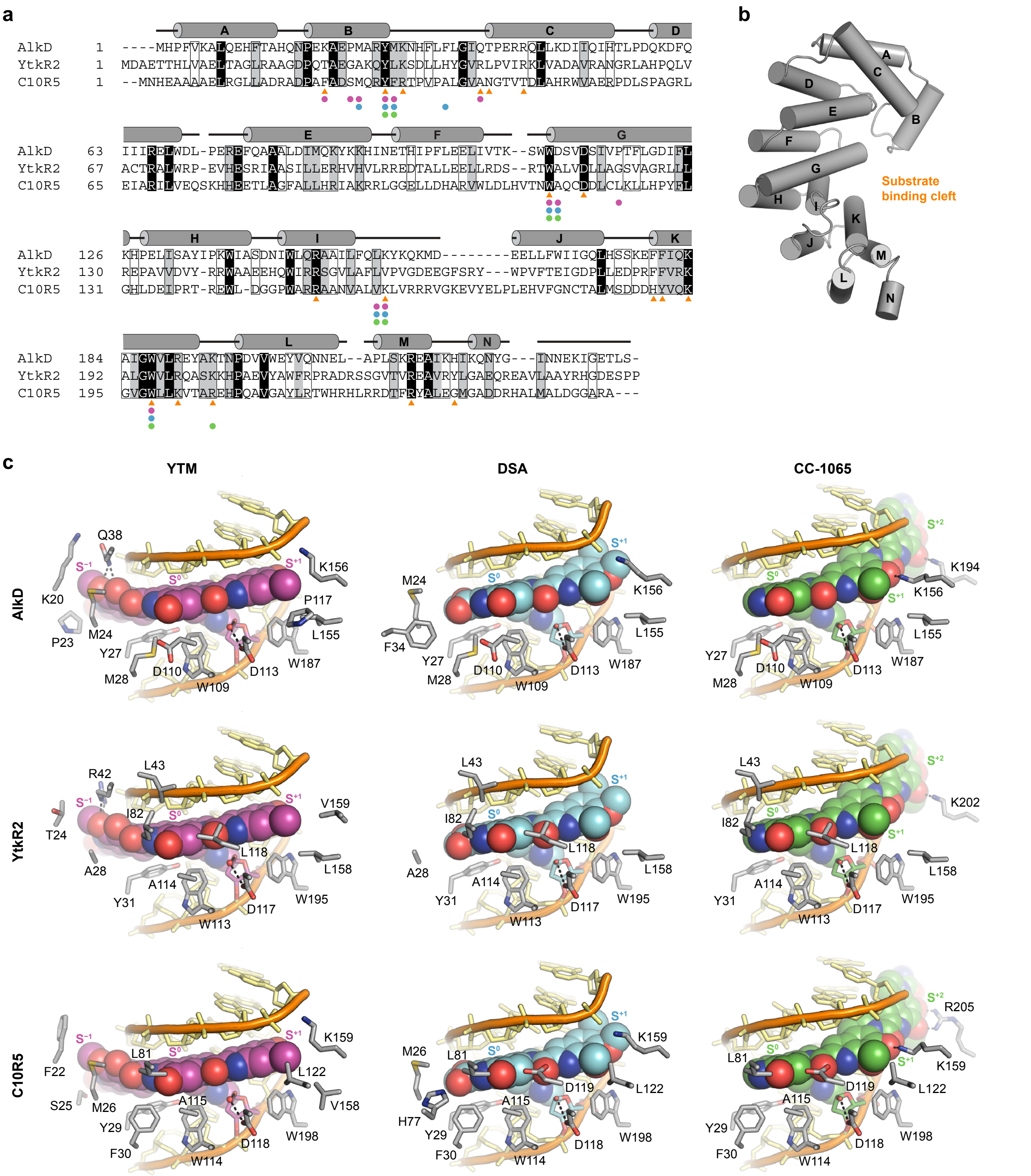

Supplementary Figure 9. Substrate specificity determinants in AlkD, YtkR2, and C10R5. (**a**) Multiple sequence alignment created with Clustal Omega. DNA binding residues in AlkD are indicated with orange triangles. YTM-Ade, DSA-Ade, and CC1065-Ade binding residues in AlkD are indicated with magenta, cyan, and green circles, respectively. Residues with non-hydrogen atoms within 5 Å of the DNA or the adducts were determined to be binding residues. Note: not all DNA binding residues interact with DNA in all complexes, and Arg215 only interacts with DNA constructs that are longer than those used in this study. (**b**) Tertiary structure of AlkD (PDB accession 5UUG). Helices are labeled as in **a**. (**c**) Close-up views of AlkD, YtkR2, and C10R5 bound to AP-DNA and each of the SCPCHD adducts. Homology models of YtkR2 and C10R5 were generated from the corresponding AlkD product complexes using SWISS-MODEL. Hydrogen-bonding interactions are indicated with dashed lines.

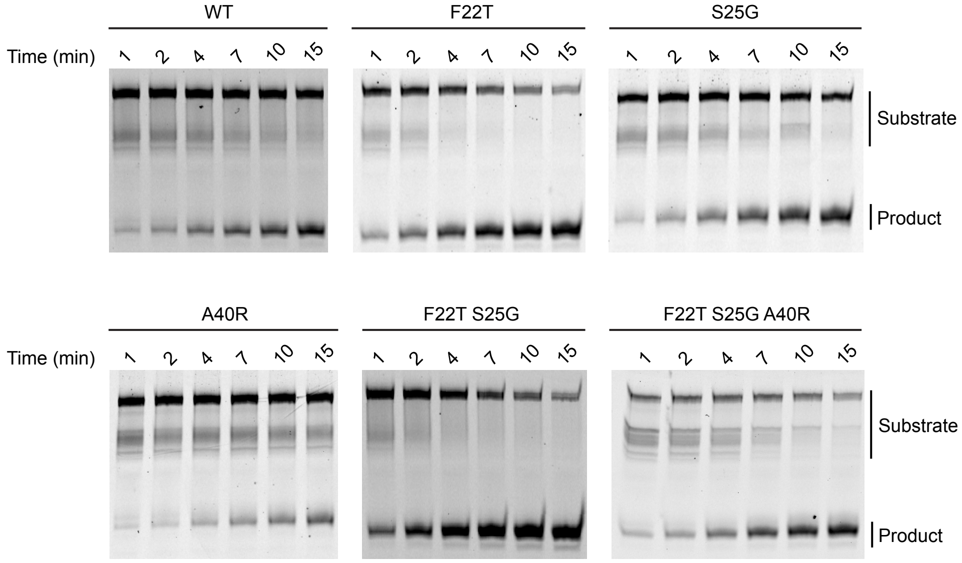

Supplementary Figure 10. Single-turnover excision of YTM-Ade by C10R5 mutants. Reactions contained 1 μM enzyme and 100 nM DNA. Full-length DNA substrate (25-mer) and cleaved product (12- or 13-mer), generated by alkaline cleavage of the AP site, were separated by denaturing gel electrophoresis. The high melting temperature of the GC-rich substrate prevented complete denaturation of the DNA duplex, producing a smeared substrate band. Experiments were performed in triplicate.

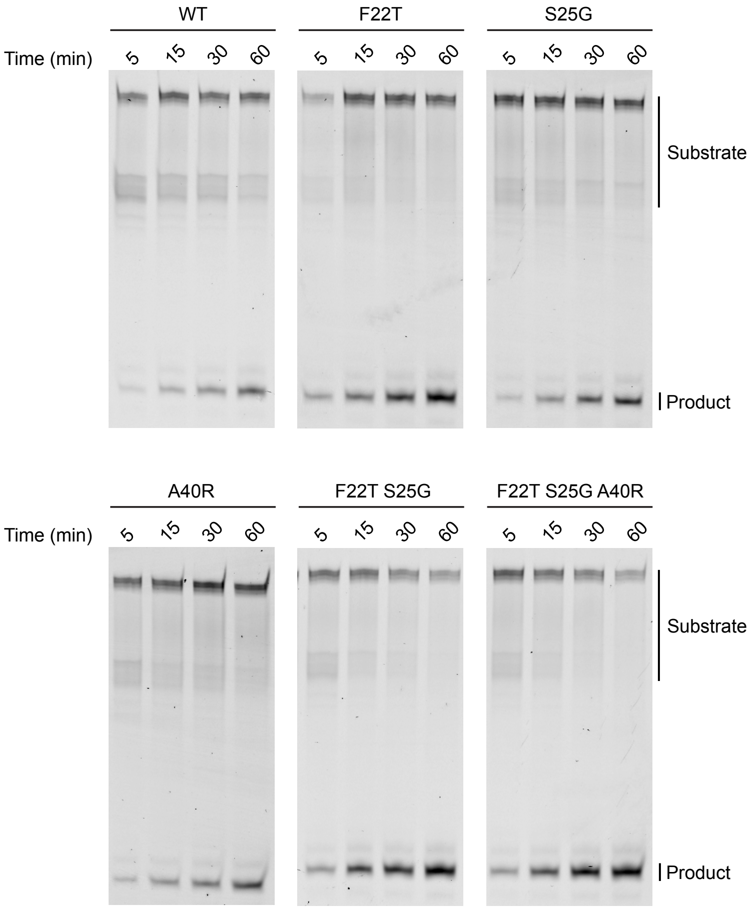

Supplementary Figure 11. Multiple-turnover excision of YTM-Ade by C10R5 mutants. Reactions contained 10 nM enzyme and 100 nM DNA. Full-length DNA substrate (25-mer) and cleaved product (12- or 13-mer), generated by alkaline cleavage of the AP site, were separated by denaturing gel electrophoresis. The high melting temperature of the GC-rich substrate prevented complete denaturation of the DNA duplex, producing a smeared substrate band. Experiments were performed in triplicate.

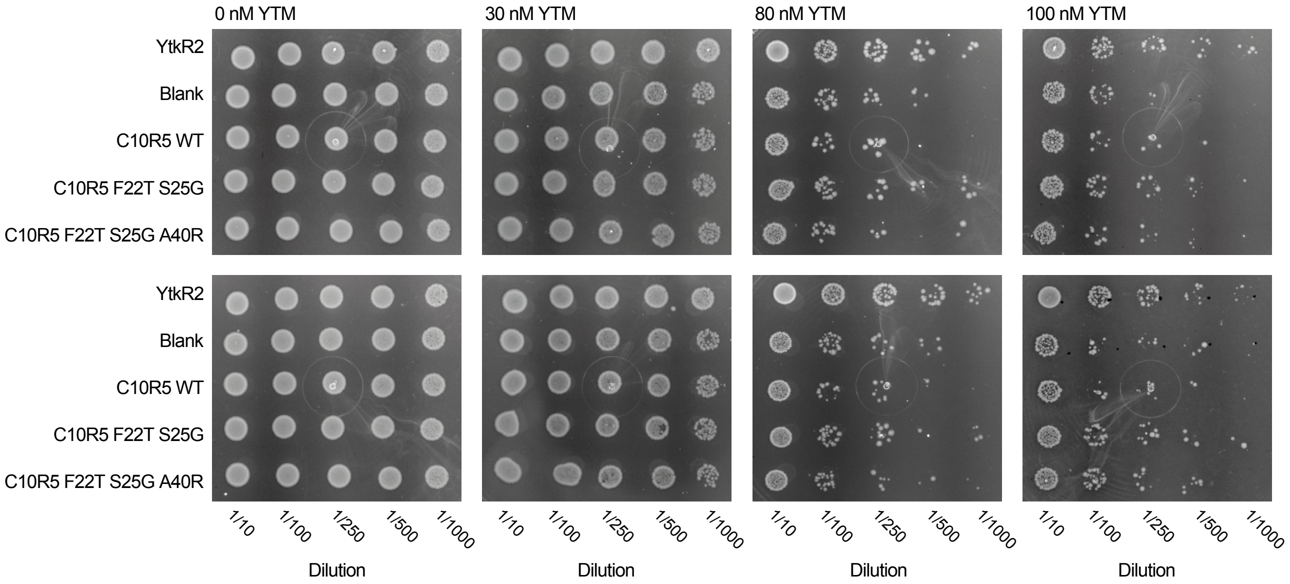

Supplementary Figure 12. YTM resistance conferred by C10R5 mutants. *E. coli* BL21(DE3) cells were transformed with either an empty plasmid (blank) or a plasmid encoding YtkR2, C10R5, or one of two C10R5 mutants and grown in the presence of various concentrations of YTM. Experiments were performed in duplicate.

Supplementary Table 1. Adduct excision rates.

Enzyme	Substrate	k_{st}* (s^{-1})	k_{mt}† (s^{-1})
AlkD	YTM-DNA	$(4.5 \pm 0.9) \times 10^{-1}$	slow
AlkD	DSA-DNA	$(2.7 \pm 0.2) \times 10^{-1}$	$(3.3 \pm 0.5) \times 10^{-5}$
AlkD	CC1065-DNA	$(2.7 \pm 0.3) \times 10^{-1}$	$(2.1 \pm 0.5) \times 10^{-5}$
YtkR2	YTM-DNA	$(5.2 \pm 0.6) \times 10^{-1}$	$(6.3 \pm 0.8) \times 10^{-4}$
YtkR2	DSA-DNA	$(2.3 \pm 0.1) \times 10^{-1}$	$(9.3 \pm 2.7) \times 10^{-4}$
YtkR2	CC1065-DNA	$(1.6 \pm 0.2) \times 10^{-1}$	$(5.5 \pm 1.7) \times 10^{-4}$
C10R5	YTM-DNA	$(1.1 \pm 0.1) \times 10^{-3}$	slow
C10R5	DSA-DNA	$(3.3 \pm 0.9) \times 10^{-2}$	$(3.6 \pm 1.5) \times 10^{-2}$
C10R5	CC1065-DNA	$(1.9 \pm 0.4) \times 10^{-2}$	$(1.1 \pm 0.2) \times 10^{-2}$

Data are presented as the mean ± SD (n=3 biologically independent experiments). Source data are provided as a Source Data file.

*k_{st} is the rate of adduct excision under single-turnover conditions (1 µM enzyme and 100 nM DNA). With all substrates, AlkD and YtkR2 approached the endpoint of the reaction by the first time point, suggesting the k_{st} values reported for these enzymes are lower limits for the actual rates.

†k_{mt} is the rate of adduct excision under multiple-turnover conditions (10 nM enzyme and 100 nM DNA). With YTM-DNA, the slow rates of excision supported by AlkD and C10R5 were insufficient for accurate determination of k_{mt} values ($< 2.1 \times 10^{-5}$ s^{-1}).

Supplementary Table 2. X-ray data collection and refinement statistics.

	AlkD/AP-DNA/DSA-Ade	AlkD/AP-DNA/CC1065-Ade
**Data collection**		
Space group	*C*2	*C*2
Cell dimensions		
a, *b*, *c* (Å)	124.72, 55.66, 47.99	124.57, 55.61, 48.56
α, *β*, *γ* (°)	90.00, 112.20, 90.00	90.00, 110.73, 90.00
Resolution (Å)	50.00–1.94 (2.01–1.94)*	50.00–1.67 (1.70–1.67)
R_{sym}	0.067 (0.598)	0.068 (0.460)
Avg. $I/\sigma I$	24.7 (2.3)	25.5 (2.7)
Completeness (%)	99.9 (98.8)	98.8 (91.9)
Redundancy	6.3 (5.9)	6.3 (4.9)
**Refinement**		
Resolution (Å)	44.45–1.93 (2.02–1.93)	31.85–1.67 (1.71–1.67)
No. reflections	22,575 (2,408)	35,843 (2,496)
R_{work}	0.154 (0.236)	0.149 (0.219)
R_{free}†	0.201 (0.290)	0.183 (0.265)
No. atoms‡		
Protein	1,929	1,929
DNA	354	354
Adduct	45	62
Water	189	357
Buffer	2	2
Avg. *B*-factors‡,§ (Å^2)		
Protein	39.8	28.6
DNA	48.5	26.7
Adduct	48.8	20.8
Water	41.7	36.3
Buffer	48.7	24.8
R.m.s. deviations		
Bond lengths (Å)	0.011	0.010
Bond angles (°)	1.116	1.067
Ramachandran distribution (%)		
Favored	96.9	96.9
Allowed	3.1	3.1
Disallowed	0.0	0.0

*Statistics for the highest resolution shell are shown in parentheses.

†R_{free} was determined from the 5% of reflections excluded from refinement.

‡Riding hydrogen atoms were not included in no. atoms or avg. *B*-factors.

§Equivalent isotropic *B*-factors were calculated in conjunction with TLS-derived anisotropic *B*-factors.

Supplementary Table 3. YTM-Ade excision rates (C10R5 mutants).

**C10R5**	$\boldsymbol{k_{st}}$* ($\mathbf{s^{-1}}$)	$\boldsymbol{k_{st}/k_{st,WT}}$†	$\boldsymbol{k_{mt}}$‡ ($\mathbf{s^{-1}}$)	$\boldsymbol{k_{mt}/k_{mt,WT}}$§
WT	$(6.9 \pm 3.3) \times 10^{-4}$	1.0	$(1.1 \pm 0.6) \times 10^{-4}$	1.0
F22T	$(3.3 \pm 0.1) \times 10^{-3}$	4.8	$(7.3 \pm 1.4) \times 10^{-4}$	6.9
S25G	$(1.1 \pm 0.1) \times 10^{-3}$	1.5	$(2.8 \pm 0.8) \times 10^{-4}$	2.6
A40R	$(2.6 \pm 0.3) \times 10^{-4}$	0.4	$(1.9 \pm 0.1) \times 10^{-4}$	1.8
F22T S25G	$(4.3 \pm 0.7) \times 10^{-3}$	6.2	$(9.8 \pm 1.7) \times 10^{-4}$	9.2
F22T S25G A40R	$(1.9 \pm 0.2) \times 10^{-3}$	2.7	$(5.4 \pm 0.9) \times 10^{-4}$	5.1

Data are presented as the mean ± SD (n=3 biologically independent experiments). Source data are provided as a Source Data file.

*k_{st} is the rate of YTM-Ade excision under modified single-turnover conditions (1 μM enzyme and 100 nM DNA).

†$k_{st}/k_{st,WT}$ is the single-turnover rate of YTM-Ade excision relative to the single-turnover rate of YTM-Ade excision by wild-type C10R5.

‡k_{mt} is the rate of YTM-Ade excision under modified multiple-turnover conditions (10 nM enzyme and 100 nM DNA).

§$k_{mt}/k_{mt,WT}$ is the multiple-turnover rate of YTM-Ade excision relative to the multiple-turnover rate of YTM-Ade excision by wild-type C10R5.
