# Supplementary material for: Structural evolution of a DNA repair self-resistance mechanism targeting genotoxic secondary metabolites
Source: Nat Commun. 2021 Nov 26;12:6942. doi: 10.1038/s41467-021-27284-7 (PMC8626424; doi:10.1038/s41467-021-27284-7)
Supplement: Supplementary file 3 — Reporting Summary [file 41467_2021_27284_MOESM3_ESM.pdf]

nature portfolio

Corresponding author(s): Brandt F. Eichman

Last updated by author(s): Nov 5, 2021

Reporting Summary

Nature Portfolio wishes to improve the reproducibility of the work that we publish. This form provides structure for consistency and transparency in reporting. For further information on Nature Portfolio policies, see our Editorial Policies and the Editorial Policy Checklist.

Statistics

For all statistical analyses, confirm that the following items are present in the figure legend, table legend, main text, or Methods section.

n/a	Confirmed	
☐	☒	The exact sample size (*n*) for each experimental group/condition, given as a discrete number and unit of measurement
☐	☒	A statement on whether measurements were taken from distinct samples or whether the same sample was measured repeatedly
☒	☐	The statistical test(s) used AND whether they are one- or two-sided<br>*Only common tests should be described solely by name; describe more complex techniques in the Methods section.*
☒	☐	A description of all covariates tested
☒	☐	A description of any assumptions or corrections, such as tests of normality and adjustment for multiple comparisons
☐	☒	A full description of the statistical parameters including central tendency (e.g. means) or other basic estimates (e.g. regression coefficient) AND variation (e.g. standard deviation) or associated estimates of uncertainty (e.g. confidence intervals)
☒	☐	For null hypothesis testing, the test statistic (e.g. *F*, *t*, *r*) with confidence intervals, effect sizes, degrees of freedom and *P* value noted<br>*Give P values as exact values whenever suitable.*
☒	☐	For Bayesian analysis, information on the choice of priors and Markov chain Monte Carlo settings
☒	☐	For hierarchical and complex designs, identification of the appropriate level for tests and full reporting of outcomes
☒	☐	Estimates of effect sizes (e.g. Cohen's *d*, Pearson's *r*), indicating how they were calculated

Our web collection on statistics for biologists contains articles on many of the points above.

Software and code

Policy information about availability of computer code

Data collection	X-ray diffraction data were collected using HKL2000 v717 (HKL Research) and an unnamed web-based interface administered by LS-CAT. Cell growth in 96-well plates was monitored using Gen5 v2.0 (BioTek).
Data analysis	Fluorescence intensity was measured with ImageQuant TL v7.0 (GE Healthcare). Regression analysis was performed with Prism v9.2 (GraphPad). X-ray diffraction data were processed using HKL2000 v717 (HKL Research). Atomic models were built and refined with Coot v0.8 and PHENIX v1.14 and validated with MolProbity v4.4. Multiple-sequence alignments were generated with Clustal v2.1. Homology models were created with SWISS-MODEL (ProMod3) v1.2. X-ray crystal structures and homology models were compared using PyMOL v1.8 (Schrödinger).

For manuscripts utilizing custom algorithms or software that are central to the research but not yet described in published literature, software must be made available to editors and reviewers. We strongly encourage code deposition in a community repository (e.g. GitHub). See the Nature Portfolio guidelines for submitting code & software for further information.

Data

Policy information about availability of data

All manuscripts must include a data availability statement. This statement should provide the following information, where applicable:

- Accession codes, unique identifiers, or web links for publicly available datasets
- A description of any restrictions on data availability
- For clinical datasets or third party data, please ensure that the statement adheres to our policy

Atomic coordinates and structure factors generated in this study were deposited in the Protein Data Bank under accession codes 7LXJ (AlkD/AP-DNA/DSA-Ade) and 7LXH (AlkD/AP-DNA/CC1065-Ade). Previously published structures of AlkD, AlkD/AP-DNA/YTM-Ade, and DSA-DNA are available in the Protein Data Bank under

accession codes 3BVS, 5UUG, and 1DSA, respectively. Source data are provided with this paper.

Field-specific reporting

Please select the one below that is the best fit for your research. If you are not sure, read the appropriate sections before making your selection.

- [x] Life sciences
- [ ] Behavioural & social sciences
- [ ] Ecological, evolutionary & environmental sciences

For a reference copy of the document with all sections, see nature.com/documents/nr-reporting-summary-flat.pdf

Life sciences study design

All studies must disclose on these points even when the disclosure is negative.

Sample size	Sample size was predetermined based on standard practices in the field. No statistical analysis was performed to select sample size.
Data exclusions	No data were excluded.
Replication	Reproducibility was ensured by performing experiments in duplicate, triplicate, or quadruplicate.
Randomization	Bacterial cells were randomly selected from precultures and randomly assigned to groups for drug resistance experiments.
Blinding	Investigators were blinded to the group allocation of any individual bacterial cell.

Reporting for specific materials, systems and methods

We require information from authors about some types of materials, experimental systems and methods used in many studies. Here, indicate whether each material, system or method listed is relevant to your study. If you are not sure if a list item applies to your research, read the appropriate section before selecting a response.

Materials & experimental systems

n/a	Involved in the study	
[ ]	[x]	Antibodies
[x]	[ ]	Eukaryotic cell lines
[x]	[ ]	Palaeontology and archaeology
[x]	[ ]	Animals and other organisms
[x]	[ ]	Human research participants
[x]	[ ]	Clinical data
[x]	[ ]	Dual use research of concern

Methods

n/a	Involved in the study	
[x]	[ ]	ChIP-seq
[x]	[ ]	Flow cytometry
[x]	[ ]	MRI-based neuroimaging

Antibodies

Antibodies used	Monoclonal mouse anti-hexahistidine primary antibody (Abgent, Catalog #AM1010A, Clone name 6AT18, Lot #unknown); goat anti-mouse secondary antibody conjugated to calf intestinal alkaline phosphatase (Cell Signaling Technology, Catalog #7056, Lot #19)
Validation	The primary antibody was validated by the manufacturer: "HIS-tagged recombinant protein was expressed in E. coli and used to test the 6xHIS monoclonal antibody (6AT18)."
